# Injury-Related Emergency Medical Service Calls, Traffic Accidents, and Crime in Mexico City Before and During the COVID-19 Pandemic

**DOI:** 10.1017/S1049023X22002230

**Published:** 2023-02

**Authors:** Esmeralda Melgoza, Hiram Beltrán-Sánchez, Arturo Vargas Bustamante

**Affiliations:** 1. Jonathan and Karin Fielding School of Public Health, University of California-Los Angeles (UCLA), Los Angeles, California USA; 2. California Center for Population Research, University of California-Los Angeles (UCLA), Los Angeles, California USA

**Keywords:** crime, Emergency Medical Services, injuries, Mexico City, traffic accidents

## Abstract

**Introduction:**

The coronavirus disease 2019 (COVID-19) pandemic had detrimental impacts across multiple sectors of the Mexican health care system. The prehospital care system, however, remains largely under-studied. The first objective of this study was to calculate the monthly per capita rates of injury-related 9-1-1 calls, traffic accidents, and crime at the state-level (Mexico City) during the early pandemic period (January 1 through June 30, 2020), while the second objective was to conduct these calculations at the borough-level for the same outcomes and time period. The third objective was to compare monthly per capita rates of injury-related 9-1-1 calls, traffic accidents, and crime at the state-level (Mexico City) during the pre-pandemic (January 1 through June 30, 2019), early pandemic (January 1 through June 30, 2020), and later pandemic periods (January 1 through June 30, 2021).

**Methods:**

A retrospective analysis was conducted to examine injury-related 9-1-1 calls, traffic accidents, and crime at the state-level (Mexico City) and borough-levels. Monthly per capita rates were calculated using four datasets, including Mexico City’s Public Release 9-1-1 Emergency Calls, National Institute of Statistics and Geography’s (INEGI) Traffic Accidents Micro-Dataset, Mexico City’s Attorney General’s Office Crime Dataset, and Projections of the Population of the Municipalities of Mexico, 2015 to 2030. All statistical analyses were conducted using STATA 17.0.

**Results:**

During the early pandemic period, injury-related 9-1-1 emergency calls, traffic accidents, and crime experienced similar trends in monthly per capita rates at the state-level and borough-levels. While the monthly per capita rates remained constant from January to March 2020, starting in March, there was a precipitous decrease across all three outcomes, although decline rates varied across boroughs. The monthly per capita rates across the three outcomes were higher during the pre-pandemic period compared to the early pandemic period. As the COVID-19 pandemic progressed, the monthly per capita rates during the later pandemic period increased across the three outcomes compared to the early pandemic period, although they did not reach pre-pandemic levels during the study period.

**Conclusion:**

The precipitous decline in injury-related 9-1-1 calls, traffic accidents, and crime in Mexico City occurred at the same time as the issuance of the first wave of public health orders in March 2020. The largest decrease across the three outcomes occurred one to two months post-issuance of the orders.

## Introduction

The first case of coronavirus disease 2019 (COVID-19) in Mexico was detected in Mexico City on February 27, 2020.^1^ Less than one month later, on March 18, 2020, Mexico City reported the first confirmed COVID-19 death.^2^ The rapidly increasing number of COVID-19 cases and deaths resulted in the issuance of several public health orders by the Mexican federal government in late March 2020.^2^ The purpose of the public health orders was to prevent new infections and mitigate the impacts of COVID-19.^2^ On March 23, 2020, the Mexican federal government ordered the closing of all schools.^2,3^ The Mexican Ministry of Health (Guerrero, Mexico) also launched *Sana Distancia*, a program that initially encouraged physical distancing, although the term was later replaced by social distancing to emphasize the importance of staying physically apart, but socially connected.^2^ On March 30, 2020, Mexico declared COVID-19 a national emergency, which resulted in the adoption of additional public health orders, including stay-at-home orders and partial suspension of non-essential activities, including leisure activities, public gatherings of more than 25 people, and public administration services.^2,4^ A total lockdown prohibiting any remaining non-essential activities was issued on April 21, 2020.^4^

The COVID-19 pandemic and the issuance of public health orders resulted in people spending more time at home, disruptions in daily activities, such as driving, and mobility restrictions, including decreases in the availability of public transportation services.^2,4,5^ At the national level, Mexico experienced a rapid decrease in the rates of traffic accidents, traffic injuries, and traffic fatalities, a finding that was, at least partially, influenced by more people staying at home.^6^ The severity of traffic accidents, however, rose after the COVID-19 pandemic began.^6^ Crimes also decreased substantially in Mexico during the COVID-19 pandemic, although variation was reported by type of crime.^7^ The reduction in crime was partially attributed to changes in mobility, including decreases in public transportation availability and public transport passenger numbers.^7,8^

The Mexican health care system was also drastically impacted by the COVID-19 pandemic and the issuance of public health orders.^9,10^ A study reported that the Mexican Institute of Social Security (IMSS; Torreón, Mexico), Mexico’s largest national public health care institution, saw a decline of 8.74 million patient visits during the early COVID-19 pandemic period, January 2019 through December 2020.^10^ As the COVID-19 pandemic progressed, in 2021, the use of IMSS health care services gradually increased.^11^ The COVID-19 pandemic was also associated with a decline in specialized care, including organ donations, waiting list additions, and organ transplantations.^12^

Although research shows that the COVID-19 pandemic had detrimental impacts across the Mexican health care system, the prehospital care system remains largely under-studied. Prehospital care refers to health care services provided in an out-of-hospital setting, usually by emergency service personnel such as emergency medical technicians and paramedics. The prehospital care system serves as an important entry point into the Mexican health care system and provides a source of real-time data, which may be used to inform the COVID-19 response.^13^ The declaration of 9-1-1 as the unified emergency number in Mexico since November 2014 also facilitates prehospital care research, although its implementation has varied by state.^14^ Mexico City, for example, began using 9-1-1 as the single emergency number starting in January 2017.^14^ Prior to the establishment of 9-1-1 as the unified emergency number in Mexico, accessing emergency services required dialing different telephone numbers based on the type of emergency.^14^ The unified 9-1-1 emergency number in Mexico standardizes the approach to emergency service data collection, which facilitates prehospital care research.

This study focuses on prehospital care in Mexico City by examining injury-related 9-1-1 emergency calls before (January 1, 2019 through June 30, 2019) and during the COVID-19 pandemic (January 1 through June 30, 2020 and 2021). Previous studies suggest decreases in injury-related 9-1-1 emergency calls, although this is the first study to examine this outcome in Mexico City.^15,16^ Furthermore, existing studies on injury-related 9-1-1 emergency calls limit their analysis to 2020, the first year of the COVID-19 pandemic, while this study expands the analysis to the first six months of 2021.^15,16^ In this study, traffic accidents and crime are examined because these incidents account for over one-half of the injuries in Mexico City.^17^ Previous studies have reported steep declines in traffic accidents and crime in Mexico City starting in March 2020, which may partially explain a decrease in injury-related 9-1-1 emergency calls during this time.^6^ This study adds to existing research on traffic accidents and crime because it examines the data at both the state-level (Mexico City; Appendix A – available online only) and the borough-level (Appendix B – available online only). This study has three objectives. The first objective is to examine monthly per capita rates of injury-related 9-1-1 calls, traffic accidents, and crime at the state-level (Mexico City) during the early pandemic period (January 1 through June 30, 2020). The second objective is to assess monthly per capita rates at the borough-level for the same outcomes and time period as the first objective. The first two objectives focus on the early pandemic period to consider changes in the monthly per capita rates across the three outcomes, before, immediately after, and three months post-issuance of the first major wave of public health orders in Mexico City in March 2020. The third objective examines monthly per capita rates for the three outcomes of interest at the state-level (Mexico City) during the pre-pandemic (January 1 through June 30, 2019), early pandemic (January 1 through June 30, 2020), and later pandemic periods (January 1 through June 30, 2021).

## Methods

Mexico City’s Public Release 9-1-1 Emergency Calls Dataset was used to examine injury-related 9-1-1 calls from January 1 through June 30, 2019, 2020, and 2021.^18^ The public dataset contains 9-1-1 emergency call data for the state (Mexico City) and its 16 boroughs.^14^ The public dataset includes all 9-1-1 emergency calls that have been sealed with a closing code, which is the last administrative step required to close a record. The lead author also submitted a freedom of information request to Mexican authorities to obtain non-publicly available socio-demographic data on patients who utilized 9-1-1 emergency services in Mexico City. Mexican authorities stated that socio-demographic patient data were unavailable. Institutional Review Board approval from the University of California, Los Angeles (UCLA; Los Angeles, California USA), Office of Human Research Protection Program was not required since the study used de-identified and publicly available datasets.

Traffic accident data were obtained from the National Institute of Statistics and Geography’s (INEGI; Aguascalientes, Mexico) Traffic Accidents in Urban and Suburban Zones Micro-Datasets. Crime data were obtained from Mexico City’s Attorney General’s Office. Traffic accident and crime data were included in the study if they were recorded from January 1 through June 30, 2019, 2020, and 2021. Traffic accident and crime data were examined at the state-level (Mexico City) and borough-levels. Lastly, to calculate monthly rates for injury-related 9-1-1 emergency calls, traffic accidents, and crime at the state-level and borough-levels, population sizes were estimated by month and borough using population counts from The Projections of the Population of the Municipalities of Mexico, 2015-2030 Dataset (Appendix C – available online only).

### Five-Step Model to Collect 9-1-1 Emergency Call Data in Mexico City

A five-step model informs the collection of 9-1-1 emergency call data in Mexico City. First, a telephone operator receives a 9-1-1 emergency call requesting care. Second, the telephone operator creates a unique incident report for each 9-1-1 emergency call. The telephone operator then collects information from the caller, including what happened, where and why the incident happened, and who was involved. Based on the information collected, the telephone operator broadly classifies the incident into one of six categories: crime, non-medical emergency, medical emergency, service call, civic concern, or false alarm. The telephone operator also categorizes the 9-1-1 call into one of 311 specific types of incidents. For example, a 9-1-1 call may be classified as a medical emergency but be categorized as an injury. Third, the telephone operator transfers the call to the Center of Command, Control, Computing, Communication, and Citizen Contact. In most cases, the first unit to be dispatched to the scene is law enforcement. Fourth, law enforcement confirms the incident and requests back-up from the appropriate agencies. Fifth, the incident classification and categorization are updated, if needed, based on the most recent facts from the responding agencies. The unique incident report is assigned a closing code by the Secretariat of Citizen Security, which is the last administrative step required to seal the record for the 9-1-1 emergency call.

### Data Analysis

A descriptive, retrospective analysis was conducted for injury-related 9-1-1 emergency calls, traffic accidents, and crimes in Mexico City and its 16 boroughs during the three periods of interest: January 1 through June 30, 2019 (pre-pandemic period), January 1 through June 30, 2020 (early pandemic period), and January 1 through June 30, 2021 (later pandemic period). All 9-1-1 emergency calls were included in the analysis if they were classified as injury-related by the time the file was sealed with a closing code. The complete date of file creation variable was used as a proxy for the date of the 9-1-1 emergency call since the published protocol instructs telephone operators to create a file during the live 9-1-1 emergency call to collect data from the caller. Traffic accident data and crime data were also included in the analysis if the event occurred in Mexico City between the three time periods of interest: pre-pandemic, early pandemic, and later pandemic.

Monthly per capita rates were calculated for injury-related 9-1-1 calls, traffic accidents, and crimes at the state-level (Mexico City) and borough-levels. Monthly per capita rates were reported instead of absolute numbers to account for differences in population sizes across Mexico City’s 16 boroughs. The INEGI provided annual population data at the state-level and borough-levels, although demographic methods were used to estimate the population per month for each borough since these data were not available (Appendix C). First, the total population for each of Mexico City’s 16 boroughs was calculated using the Projections of the Population of the Municipalities of Mexico, 2015-2030 Dataset. Second, the mean annualized growth rate was computed. Third, the growth rate was applied to predict population sizes by month for each borough. Fourth, the population sizes by month for each borough served as the denominator to calculate the rate for injury-related 9-1-1 calls, traffic accidents, and crimes. Fifth, the monthly per capita rates were multiplied by 100,000 for clarity of interpretation at the borough-level. Sixth, Mexico City-level rates for injury-related 9-1-1 calls, traffic accidents, and crimes were calculated using the borough-level rates for each of the three outcomes of interest (Appendix C). All statistical analysis was conducted using STATA, version 17.0 (STATACorp LLC; College Station, Texas USA).

## Results

**Figure 1.**
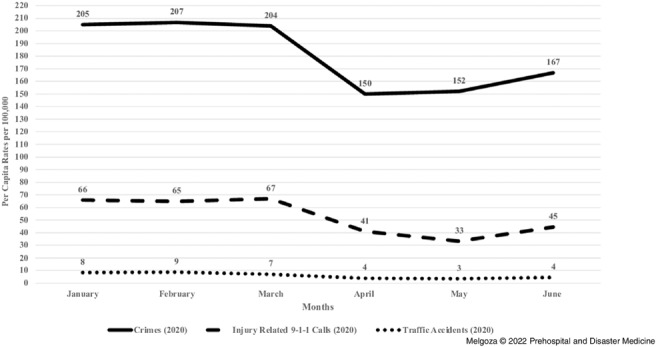
Rates of Crime, Injury-Related 9-1-1 Calls and Traffic Accidents in Mexico City from January 1 through June 30, 2020.

The first objective of this study was to examine per capita rates of injury-related 9-1-1 emergency calls, traffic accidents, and crime at the state-level (Mexico City) from January 1, 2020 through June 30, 2020. From January 1 through June 30, 2020, monthly per capita rates of injury-related 9-1-1 calls, crimes, and traffic accidents experienced similar time trends (Figure 1). While the rates remained constant from January to March 2020, starting in March, the monthly per capita rates for injury-related 9-1-1 calls, crimes, and traffic accidents decreased precipitously with the largest decline among traffic accidents. For example, traffic accidents, injury-related 9-1-1 calls, and crimes experienced a decline of 57%, 51%, and 25.5%, respectively. The decline across all three outcomes occurred at approximately the same time as the issuance of public health orders in Mexico City in March 2020. However, the monthly per capita rates increased substantially by June 2020 across all three outcomes, although the rate was lower to that observed during the first three months of the year.

**Figure 2.**
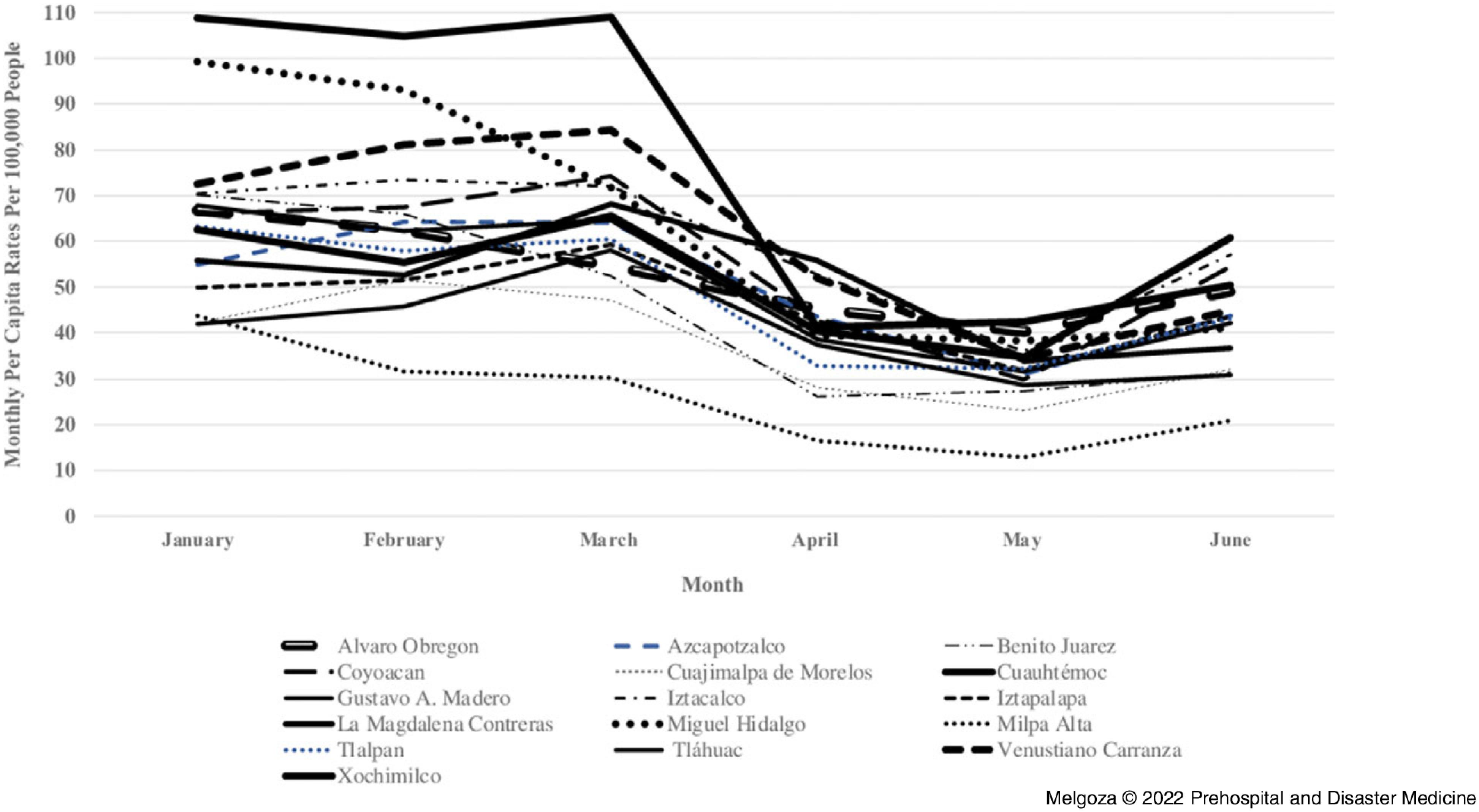
Injury-Related 9-1-1 Emergency Call Rates in Mexico City’s 16 Boroughs from January 1 through June 30, 2020.

**Figure 3.**
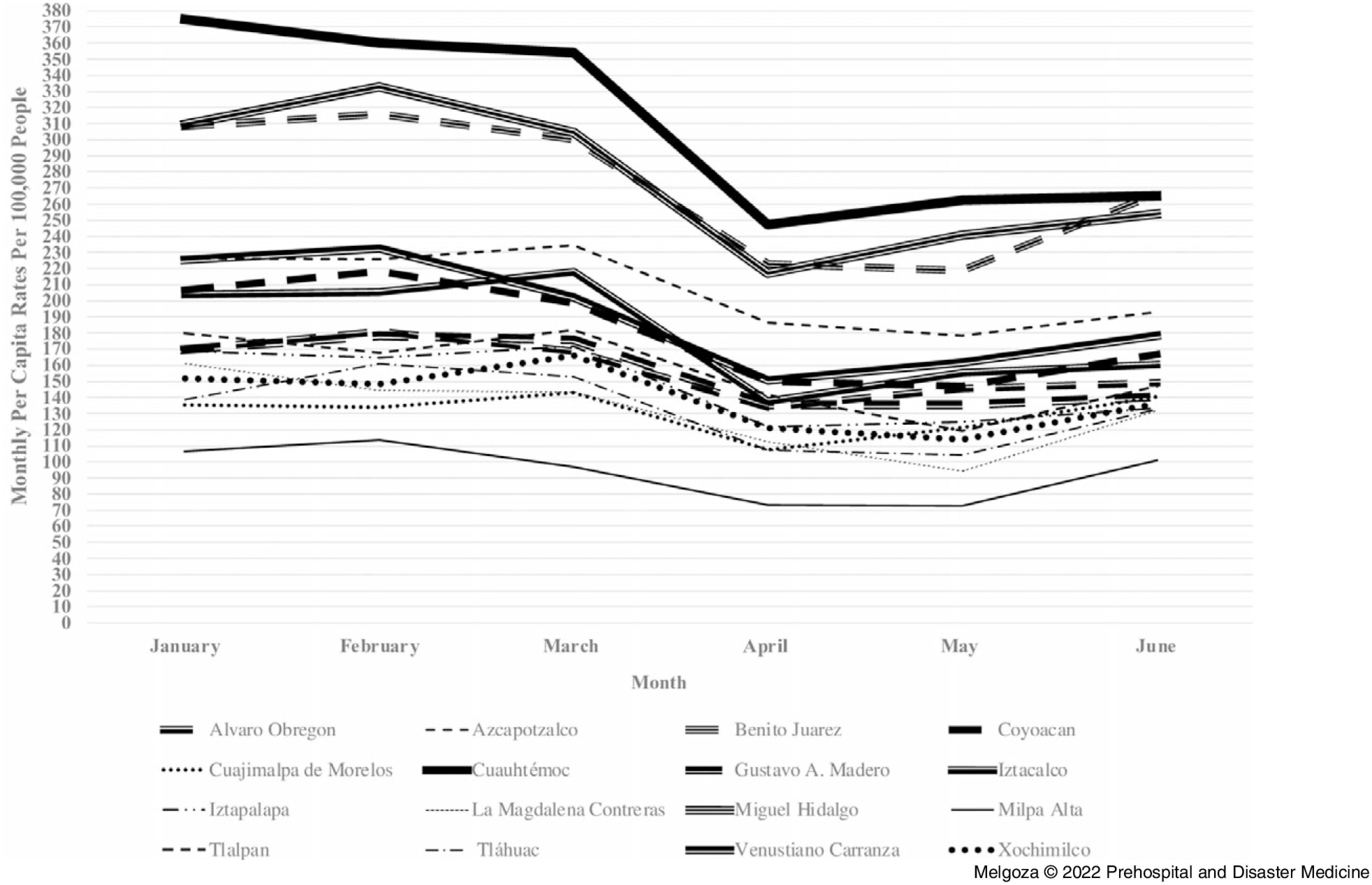
Crime Rates in Mexico City’s 16 Boroughs from January 1 through June 30, 2020.

**Figure 4.**
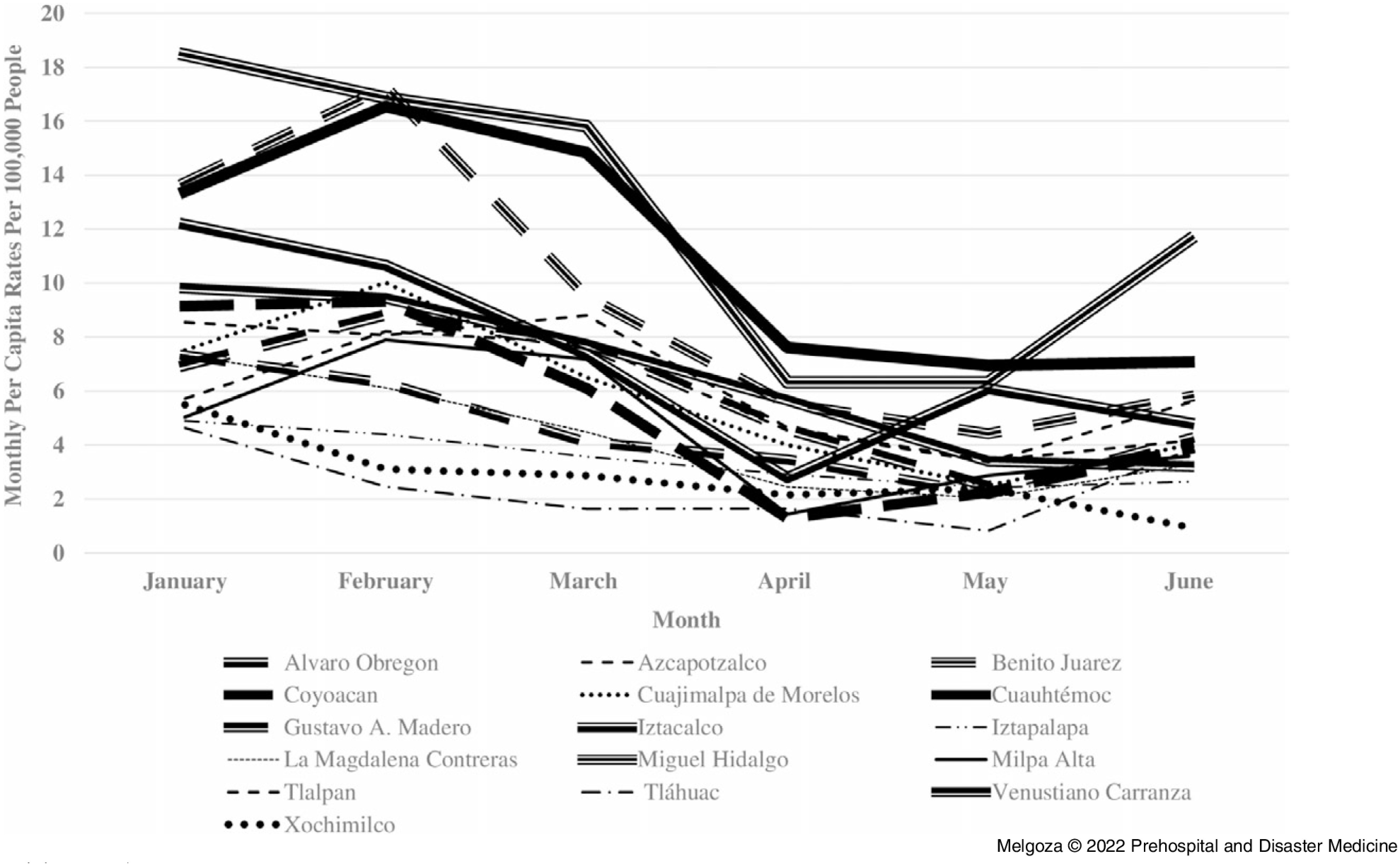
Traffic Accident Rates in Mexico City’s 16 Boroughs from January 1 through June 30, 2020.

The second objective of the study was to compare monthly per capita rates of injury-related 9-1-1 emergency calls, traffic accidents, and crime across Mexico City’s 16 boroughs during January 1 through June 30, 2020 (Figure 2, Figure 3, Figure 4). Results at the borough-level show that the rates of injury-related 9-1-1 calls, crimes, and traffic accidents showed a similar trend as that of Mexico City, although there was some variation in the rate of decline by borough, especially at the time of the first wave of public health orders in March 2020. For example, the largest decline in the rates of injury-related 9-1-1 calls from March to June 2020 occurred in Cuauhtémoc (53.9%), Tláhuac (46.9%), and Venustiano Carranza (46.8%). In contrast, the smallest decline in the rates of injury-related 9-1-1 calls during the same period occurred in Xochimilco (7.2%), Alvaro Obregon (10.1%), and Iztacalco (20.7%). Similarly, the largest decline in the rates of crime from March through June 2020 occurred in Iztacalco (26.4%), Cuauhtémoc (25.2%), and Iztapalapa (22.3%). In contrast, there was an increase in crime rates from March through June 2020 in Milpa Alta (4.4%), and the smallest decline in Cuajimalpa de Morelos (1.5%) and La Magdalena Contreras (8.2%). Lastly, the largest decline in rates of traffic accidents from March through June 2020 occurred in Xochimilco (66.6%), Venustiano Carranza (58.5%), and Azcapotzalco (52.7%). On the contrary, there was an increase in the rate of traffic accidents during the same time period in Tláhuac (133.5%) and Alvaro Obregon (3.1%). Overall, from March through June 2020, Venustiano Carranza had one of the largest declines in monthly per capita rates for both injury-related 9-1-1 calls and traffic accidents, while Cuauhtémoc had one of the largest declines in monthly per capita rates for both injury-related 9-1-1 calls and crime. Alvaro Obregon had one of the smallest declines in monthly per capita rates from March through June 2020 for both injury-related 9-1-1 calls and traffic accidents.

**Figure 5.**
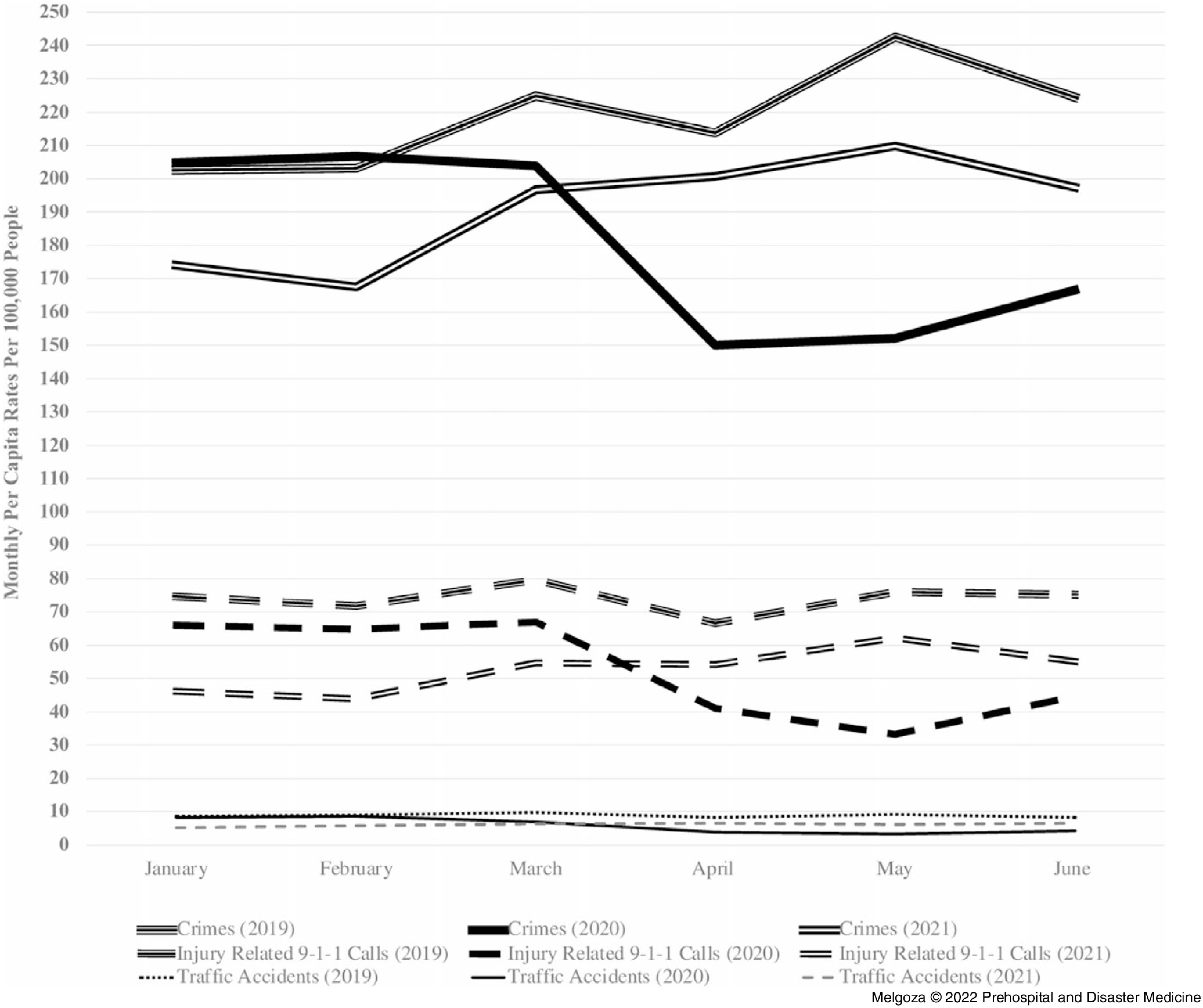
Injury-Related 9-1-1 Emergency Calls, Traffic Accidents, and Crimes in Mexico City During the Pre-Pandemic, Early Pandemic, and Later Pandemic Periods.

The third objective of this study was to compare the monthly per capita rates of injury-related 9-1-1 calls, traffic accidents, and crime in Mexico City during the three periods of interest: pre-pandemic (January 1 through June 30, 2019), early pandemic (January 1 through June 30, 2020), and later pandemic periods (January 1 through June 30, 2021). Figure 5 shows monthly per capita rates per 100,000 people grouped by outcome and pandemic period (pre-pandemic, early pandemic, and later pandemic). Results show that the monthly per capita rates of injury-related 9-1-1 calls, crime, and traffic accidents were higher during the pre-pandemic period (January 1 through June 30, 2019) and the first three months of the early pandemic period (January 1 through March 31, 2020), but they decreased precipitously starting in March 2020, which corresponds to the third month of the early pandemic period. However, during the later pandemic period, January 1 through June 30, 2021, monthly per capita rates increased again for all outcomes, although these increases did not reach pre-pandemic levels during the study period.

## Discussion

The study findings provide evidence to support the idea that the greatest impact from public health policies is not immediate. Previous studies have found that public health policies may take weeks, months, or even years to create noticeable change.^19^ In this study, the monthly per capita rates of injury-related 9-1-1 calls, traffic accidents, and crimes decreased precipitously in Mexico City starting in March 2020, which was at the same time as the issuance of the first wave of public health orders by the Mexican government. Stay-at-home orders led to more people staying at home and decreased mobility, which likely resulted in less crime, traffic accidents, and fewer injury-related 9-1-1 calls. The lowest rates of injury-related 9-1-1 calls, traffic accidents, and crime, however, were recorded one to two months post-issuance of the first wave of public health orders.

A few studies have been published that examine the prehospital care system in Mexico City.^20–22^ Even fewer studies have been published that examine the prehospital care system using borough-level analyses of Mexico City.^23^ In this study, the results at the state-level and borough-levels show similar trends for injury-related 9-1-1 calls, crime, and traffic accidents, although there is some variation in the rate of decline by borough, especially at the time of the first wave of public health orders in March 2020. The variability in the rate of decline across the three outcomes may be attributed to multiple factors that characterize each of the boroughs. Although the per capita rates account for differences in borough size, it is important to note that population size, population density, economic activities, and socio-demographic characteristics differ across Mexico City’s boroughs.^24^ The population density varies across the different boroughs, which in the context of the COVID-19 pandemic is an important consideration since overcrowding may correspond to higher risk of transmission and lower socioeconomic status. Certain economic characteristics across the 16 boroughs may influence the rate of decline for injury-related 9-1-1 calls, traffic accidents, and crime. For example, previous studies in other countries have shown that older adults are at higher risk for injuries and have higher 9-1-1 utilization rates compared to the general population.^25–27^ Although patient socio-demographic characteristics were not available in the prehospital care dataset, future research should examine associations between borough socio-demographic characteristics and injury-related 9-1-1 calls.

During the later pandemic period (January 1 through June 30, 2021), injury-related 9-1-1 emergency calls gradually increased, compared to the early pandemic period, although they remained below pre-pandemic levels. The increase in injury-related 9-1-1 emergency calls during the later pandemic period may be attributed to a myriad of factors, including less restrictive public health orders and increased access to COVID-19 vaccines, especially for high-risk groups.^28^ Mexico launched a universal and free vaccination campaign for high-risk groups, including older adults, starting in February 2021.^28^ Increased access to COVID-19 vaccines, combined with less restrictive public health orders, likely increased outdoor mobility, including driving and participation in recreational activities. In turn, these changes in people’s behavior may have contributed to the gradual increase in injury-related 9-1-1 emergency calls, traffic accidents, and crime in Mexico City during the later pandemic period.

Although this is the first study to examine injury-related 9-1-1 calls before and during the COVID-19 pandemic in Mexico City, it is important to note that more research is needed to better understand the prehospital care system. Mexico City’s prehospital care system is comprised of both public (government-operated) and private ambulances, although the data used in this study come from 9-1-1 callers who requested emergency services from the government-operated emergency system. In Mexico City, however, the public ambulance to population ratio is extremely small with 45 public ambulances servicing a city of over nine million people.^29^ The small public ambulance to population ratio is especially problematic during surges, such as the COVID-19 pandemic, because the demand is higher than the supply of prehospital care resources. One of the impacts of the small public ambulance to population ratio in Mexico City was the increase in the operation of private ambulances that were often ill equipped and operated by untrained personnel.^30^ Future efforts by the Mexican government should ensure that the supply of prehospital care resources, including trained emergency service personnel and emergency vehicles, is enough to meet the demand of the population. More investments in other sectors of the Mexican health care system may also reduce the use of prehospital care for low-acuity 9-1-1 calls. With fewer low-acuity 9-1-1 calls, prehospital care services can focus their attention on high-acuity 9-1-1 calls. Additionally, the Mexican government should also create better safeguards to protect consumers from profiteering private ambulances by creating and better enforcing standards of care.

## Limitations

First, this study uses a publicly available prehospital care dataset that includes data obtained from individuals who called the government-owned 9-1-1 emergency system. Mexico City’s prehospital care system is comprised of both public (government-operated) and private ambulances. The prehospital care data from private ambulances may not be recorded in the dataset, although the dataset that was used is the best source of data available. Second, the prehospital care dataset used in this study includes 9-1-1 emergency calls that have been sealed with a closing code. A closing code is the last required administrative step to seal a record that was created by a telephone operator. The 9-1-1 emergency calls without a closing code are not included in the study analysis. Third, the use of traffic accidents and crimes as measures of mobility during the COVID-19 pandemic could have some reporting bias. Minor traffic accidents are less likely to be recorded if no authority is requested on the scene. Similarly, under-reporting of crime is widespread in Mexico. However, the limitations to traffic accident and crime reporting are the same across all the years studied, which will likely not impact the findings. Fourth, the borough closure variable was used as a proxy to determine the geographic location of the 9-1-1 emergency call. Although an imperfect measure, the borough closure variable is the best variable available in the public dataset to determine the geographic location of the 9-1-1 emergency call. Fifth, the study uses the classification and categorization procedure that is determined by the 9-1-1 telephone operator based on the information provided by the caller. Lastly, the analysis does not use the closing code variable that labels the 9-1-1 emergency call using one of the following options: affirmative, negative, informational, duplicate, and false. The focus is on injury-related 9-1-1 emergency calls that were made by the public during the study periods, not on whether the 9-1-1 emergency calls were deemed legitimate.

## Conclusion

In this study, there was a steep decline in the rate of injury-related 9-1-1 calls, traffic accidents, and crime at both the state-level (Mexico City) and borough-levels at the same time as the issuance of the first wave of public health orders in March 2020. The largest decrease in the rates across all three outcomes, however, occurred one to two months post-issuance of the public health orders. As the COVID-19 pandemic progressed, the rates of injury-related 9-1-1 calls, traffic accidents, and crimes increased, although they did not reach pre-pandemic levels.

## Supplementary material

For supplementary material accompanying this paper visit https://doi.org/10.1017/S1049023X22002230. click here to view supplementary material
