# Supplementary material for: Injury-Related Emergency Medical Service Calls, Traffic Accidents, and Crime in Mexico City Before and During the COVID-19 Pandemic
Source: Prehosp Disaster Med. 2022 Nov 28;38(1):73–80. doi: 10.1017/S1049023X22002230 (PMC9885436; doi:10.1017/S1049023X22002230)
Supplement: Supplementary file 1 [file S1049023X22002230sup001.docx]

**Appendix A**

Source: Estados Unidos Mexicanos: Division Estatal. INEGI. <https://cuentame.inegi.org.mx/mapas/pdf/nacional/div_territorial/nacionalestados.pdf>. Accessed September 11, 2022.

**Appendix B**

Source: Ciudad de Mexico. INEGI. <https://cuentame.inegi.org.mx/mapas/pdf/entidades/div_municipal/cdmx_demarcaciones_byn.pdf>. Accessed September 11, 2022.

**Appendix C**

Calculating the monthly per capita rates of injury-related 9-1-1 calls, traffic accidents, and crime in Mexico City’s 16 boroughs.

1. Calculate the total population by borough using the Projections of the Population of the Municipalities of Mexico, 2015-2030 Dataset. This will give you the total population per year by borough.

2. Compute the mean annualized growth rate using the following equation: ln(P2/P1) and k

*P1 = Population at time 1

*P2 = Population at time 2

* k = The fraction of a year that has elapsed

3. Apply the mean annualized growth rate to predict population sizes by month for each borough.

4. Use the population sizes by month for each borough as the denominator to calculate the rate for injury-related 9-1-1 calls, traffic accidents, and crimes.

5. Multiple the per capita rates by 100,000 for clarity of interpretation

6. Mexico City-level rates for injury-related 9-1-1 calls, traffic accidents, and crimes are calculated using the borough-level rates.

7. Calculate the ratio of the borough population size divided by the total population size of Mexico City. The total population size in Mexico City in each month is the sum of population sizes across all boroughs.

8. Multiply each population ratio by the corresponding rate in each borough and sum them all. This will provide the total rate for Mexico City for January to June in each year.
